# Comprehensive Study of Phenotypic and Growth Rate Features of *Blastocystis* Subtypes 1–3 and 6 in Symptomatic and Asymptomatic Subjects

**Published:** 2019

**Authors:** Seyed Ahmad KARAMATI, Hamed MIRJALALI, Maryam NIYYATI, Tahereh REZAEI RIABI, Abbas YADEGAR, Hamid ASADZADEH AGHDAEI, Ali HAGHIGHI, Seyyed Javad SEYYED TABAEI, Mohammad Reza ZALI

**Affiliations:** 1. Department of Medical Parasitology and Mycology, School of Medicine, Shahid Beheshti University of Medical Sciences, Tehran, Iran; 2. Foodborne and Waterborne Diseases Research Center, Research Institute for Gastroenterology and Liver Diseases, Shahid Beheshti University of Medical Sciences, Tehran, Iran; 3. Basic and Molecular Epidemiology of Gastrointestinal Disorders Research Center, Research Institute for Gastroenterology and Liver Diseases, Shahid Beheshti University of Medical Sciences, Tehran, Iran; 4. Gastroenterology and Liver Diseases Research Center, Research Institute for Gastroenterology and Liver Diseases, Shahid Beheshti University of Medical Sciences, Tehran, Iran

**Keywords:** *Blastocystis*, Subtypes, Phenotypic variation, Generation time

## Abstract

**Background:**

The present study aimed to assess the grouping of subtypes 1–3 and 6 of *Blastocystis* according to the size and generation time of the parasite among the symptomatic and asymptomatic subjects.

**Methods:**

*Blastocystis* subtypes 1–3 and 6 isolated from symptomatic and asymptomatic subjects and were cultivated in DMEM medium. In order to assess inter- and intra-subtype variation in size, all the isolates were measured using morphometric criteria. Generation time was calculated using approximately 1×10^4^ *Blastocystis,* which were cultivated in DMEM, every 24h for 4 days.

**Results:**

All subtypes had 5 to 185 μm diameter range. The smallest size was attributed to ST1, followed by ST6 and ST2. ST3 showed the most variable size and phenotypes compared with the other three subtypes. Furthermore, amoeboid forms and parasite clumping were only seen in ST3-S (symptomatic subjects). Generation time analysis showed that the number of ST1 isolated from symptomatic and asymptomatic subjects peaked higher than the other subtypes.

**Conclusion:**

This is the first study discussing inter-intra-size, phenotype and generation time variations among 4 common subtypes of *Blastocystis*. Accordingly, ST3 was largest subtype and showed most diversities in both size and phenotype, while ST1 was smallest subtype with lowest intra-subtype variation.

## Introduction

*Blastocystis* is an anaerobic cosmopolitan eukaryote that infects intestinal tract of humans and broad range of animals (1–5). Although the main transmission mode of the infection has not been absolutely identified, *Blastocystis* infection occurs through ingestion of cyst by the fecal-oral route through poor hygiene practices (6–8). Prevalence of *Blastocystis* in humans usually varies according to sanitation situation among different communities (1, 9) and it was reported up to 100% among children in Senegal (10). This organism is characterized by 4 well-known forms in either stool samples or in vitro cultures including vacuolar, granular, amoeboid and cyst together with a number of less common forms (1, 11, 12) and significant differences in their size (13). However, the vacuolar form has been suggested as the most common observed morphotype in stool samples and laboratory culture (14).

Several microscopic methods including bright field microscopy of wet mount smear, light, transmission electron microscopy and smear staining have been applied to study morphological criteria of this parasite (6, 13, 15–18). However, there is not enough evidence of morphological differences based on the host. Nevertheless, because of extensive intra-subtype variations in the size of vacuolar and fecal cyst forms (9, 13), distinguishing subtypes from each other using the morphological criteria remains as a laboratory challenge (9).

Concerning the latest epidemiological studies, extensive genetic variations have seen between/within subtypes of *Blastocystis* isolated from both animals and humans (1, 19–21). Although the correlation between gastrointestinal symptoms and subtypes have not been well established, many studies have shown association between specific subtypes and some clinical manifestations (21–25). Indeed, some forms like amoeboid have mostly reported from patients who suffer from urticarial (26).

However, various subtypes of *Blastocystis* may show inter-and intra-subtype variations in the size and phenotype as well as generation time regarding the presence of symptoms. Therefore, the current research aimed to study size, morphotype variations and generation time among ST1-3 and 6 isolated from symptomatic and asymptomatic individuals.

## Methods

### Sample collection

Overall, 26 positive isolates of *Blastocystis* subtypes 1–3 and 6 obtained from symptomatic/asymptomatic individuals (without any known causative agents), transferred to the Parasitology Laboratory located at Foodborne and Waterborne Diseases Research Center (FWDRC), Research Institute for Gastroenterology and Liver Diseases (RIGLD), Shahid Beheshti University of Medical Sciences, Tehran, Iran, from Aug 2016 to Aug 2017, were included in the current study (Table 1). Although most of symptoms were non-specific, reported symptoms in the symptomatic individuals were diarrhea, constipation, bloating, vomiting, abdominal pain and nausea. All positive samples were previously subtyped using amplification and sequencing of the “barcoding region” of SSU rRNA gene (27).

**Table 1:** Subtypes and the number of each subtype included in the current study.

	***ST1***	***ST2***	***ST3***	***ST6***
Symptomatic	4	3	4	2
Asymptomatic	1	6	6	-

### Microscopic examination and in vitro culture

The samples were inoculated in Dulbecco’s modified Eagle medium (DMEM) (Gibco, Thermo Fisher Scientific, MA, USA) containing Penicillin-Streptomycin (1000-unit penicillin and 4 mg/ml streptomycin) (Gibco, Thermo Fisher Scientific, MA, USA) supplemented with 20% inactivated fetal bovine serum and were incubated at 37 °C in an anaerobic condition. The cultivated samples were examined every 48–72 h by direct microscopy and iodine staining.

### Size and phenotype variation analysis

In order to study inter-and intra-subtype variation in the size and phenotype, all relevant information of the parasite was recorded during each sub-culture. Size and morphology of all samples were assessed on 2-d-cultivated *Blastocystis*. In order to size measurement, fifty parasites were randomly chosen from 10 different portions of each slide. The mean size together with smallest and largest parasites for each subtype were calculated and entered to SPSS software for further analysis. The parasites were measured using microscope reticle scale and magnification 400X.

### Generation time variation

For assessing the generation time of each subtype, approximately 1×10^4^ parasite from a 3-day-culture of the parasite was cultivated in DMEM medium supplemented with 20% fetal bovine serum and Penicillin-Streptomycin (1000-unit penicillin and 4 mg/ml streptomycin) (Gibco, Thermo Fisher Scientific, MA, USA) as mentioned elsewhere (28). The parasite number per μl was counted and recorded by haemocytometer chamber every 24 h for 4 d. The generation times of *Blastocystis* isolates in the media were calculated based on microscopic measures (29) base on the following formula.

Tg*=(T2−T1)/(log2(n2/n1))

* Tg denotes the generation time, n1 represents the number of cultured parasitic organisms at the initial time (T1), and n2 represents the number of parasitic cells at subsequent time (T2). Thus, (T2−T1) = 24 h of *in vitro* culture. We used this formula every 24h to provide generation time during 96 h.

All the experiments were performed in duplicates to reduce the chance of errors.

### Statistical Analysis

Statistical analysis using One-Way ANOVA was performed to test the correlation between size of *Blastocystis* and subtype. The association between subtype and size variations, symptom and generation time was analyzed as well. IBM SPSS Statistics for Windows, v22 (Chicago, IL, USA) was applied to calculate statistical analysis. A probability *P-*value<0.05 was considered statistically significant.

### Ethical approval

All procedures performed in this study were in accordance with the ethical standards (IR.SBMU.RIGLD.REC.1395.84) released by Ethical Review Committee of the Research Institute for Gastroenterology and Liver Diseases, Shahid Beheshti University of Medical Sciences, Tehran, Iran.

## Results

### Microscopic descriptive analysis

**Fig. 1:**
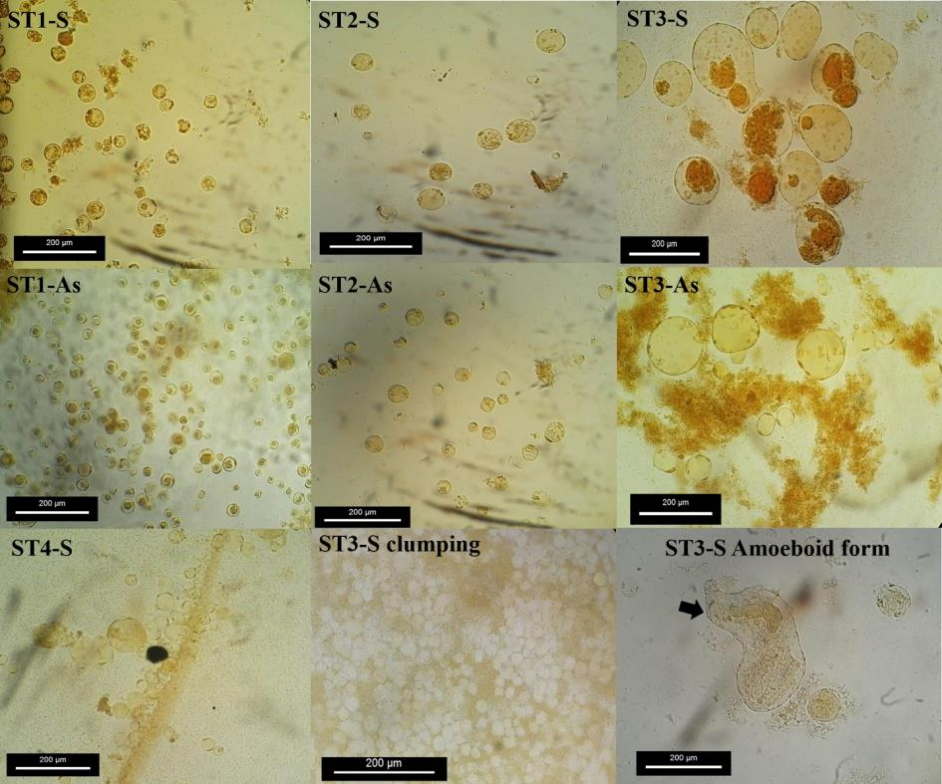
Variation size, clumping and amoeboid form of *Blastocystis* subtypes. (S: symptomatic subjects and As: asymptomatic subjects)

**Fig. 2:**
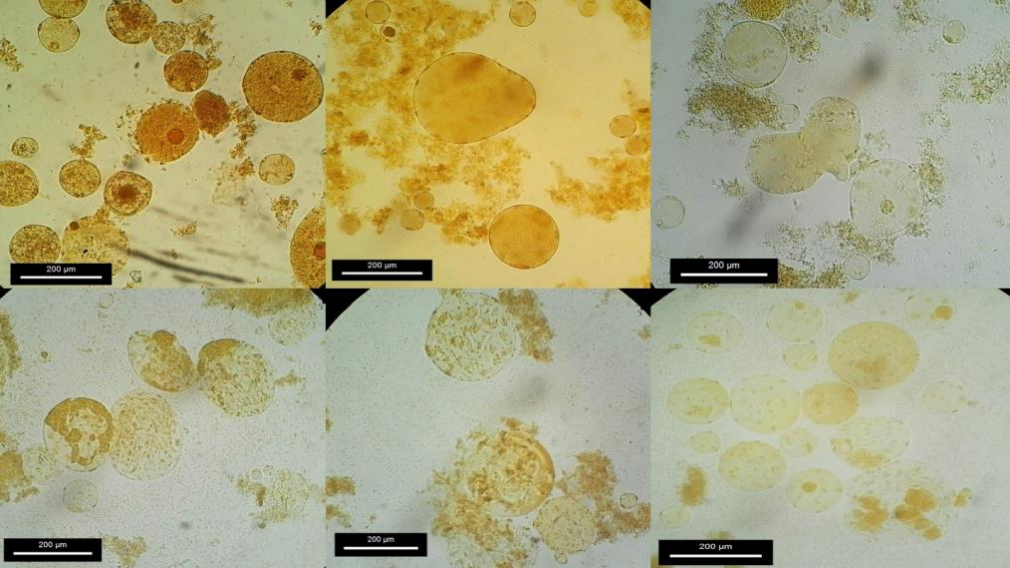
Phenotypic & size variation of *Blastocystis* ST3 isolated from symptomatic subjects

Size and morphology of the parasite were seen varied between/within subtypes. All four subtypes of the parasite were spherical and showed size range from 5 to 185 μm (mean range 17.5 μm). ST1 showed the smallest size (5–30 μm / mean: 10.42 μm), followed by ST6 (7–26 μm/mean: 15 μm), ST2 (7–89 μm/mean: 17.9 μm) and ST3 (7–185 μm/mean: 25.94 μm). The size and phenotypic variation of the parasite varied during different conditions (Fig. 1). Furthermore, ST3 showed most variation in the size and phenotypes compared with the other three subtypes (Figs. 1, 2). Amoeboid forms and remarkable clumping of the parasite were only seen in ST3 isolated from symptomatic subjects (Figs. 1, 2), while it was not observed in other three subtypes isolated from symptomatic patients and subtypes isolated from asymptomatic subjects.

**Fig. 3:**
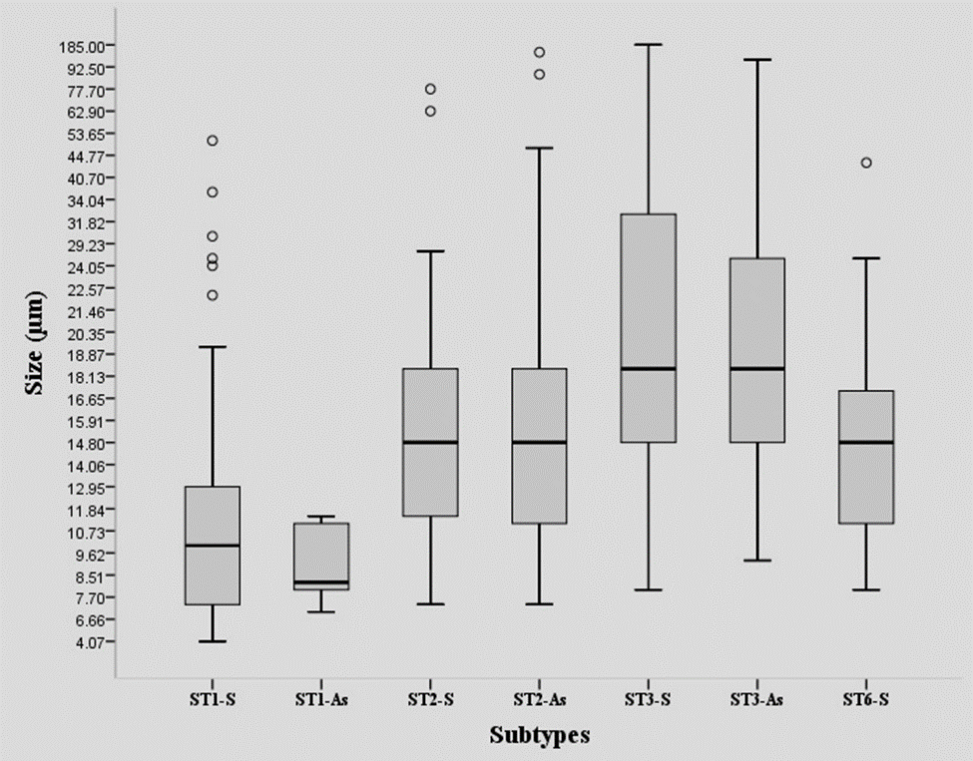
Variation size between *Blastocystis* subtypes (ST1 –3 and 6). ST3 isolated from symptomatic subjects showed significantly higher size range in comparison with other groups apart from ST3 isolated from asymptomatic subjects

The findings of size variation showed that based on one-Way ANOVA test, there was significant correlation between size and subtype (Fig. 3). ST3-S was significantly larger than other subtypes isolated from both symptomatic and asymptomatic individuals, although there was no significant difference in subtype 3 isolated from asymptomatic and symptomatic patients. Statistical analysis was also represented significant differences between subtypes, although there was not significant intra-subtype variation in size according to presence of symptoms (Fig. 3 and Table 2).

**Table 2:** *P*-values of the comparison of size variation between groups included in the analysis

***Subtypes***	***ST1-S***	***ST1-As***	***ST2-S***	***ST2-As***	***ST3-S***	***ST3-As***
ST1-S						
ST1-As	0.997					
ST2-S	0.434	0.593				
ST2-As	0.304	0.594	1.000			
ST3-S	0.000	0.001	0.002	0.000		
ST3-As	0.000	0.016	0.109	0.051	0.621	
ST6-S	0.918	0.861	0.999	0.998	0.003	0.102

### Growth characteristics

**Fig. 4:**
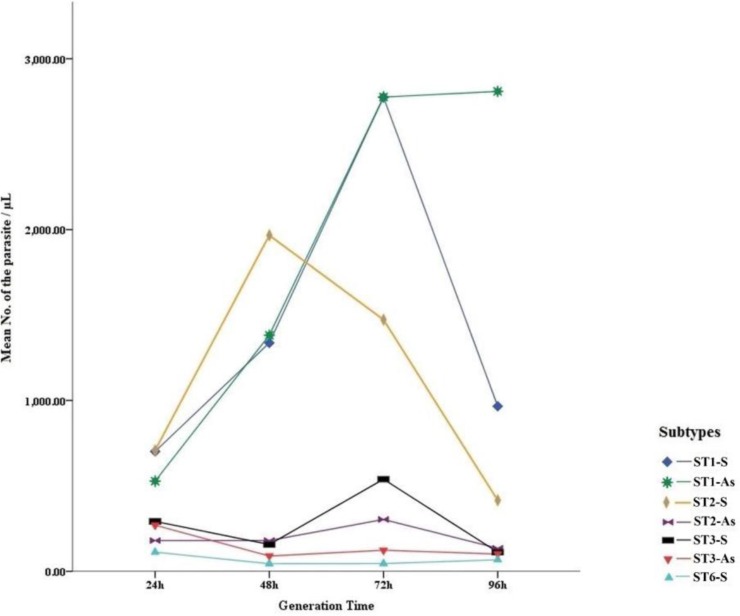
Growth profile of *Blastocystis* isolates (ST1–3 and 6) cultured in DMEM medium in four times (24–96 h)

Generally, in order to increase the accuracy of the study, two isolates from each subtype were used for calculation of generation time. Accordingly, the results represented that apart from ST2 isolated from symptomatic patients, number of the parasite of all other subtypes was peaked 72 h after cultivation. The number of *Blastocystis* ST1 isolated from symptomatic and asymptomatic subjects was more than other subtypes. Furthermore, ST6 showed lowest number of parasite compared with other subtypes (Fig. 4).

## Discussion

Despite the numerous studies, pathogenicity of *Blastocystis* is still controversial (30–33). Phenotypic differences of the parasite have been reported from symptomatic and asymptomatic cases. This finding may be related to the inter-and intra-subtype variation of *Blastocystis* (28, 30, 34). This is the first study assessing inter-and intra-subtype variations in size, phenotype and generation time of subtypes 1–3 and 6 of *Blastocystis* isolated from symptomatic and asymptomatic subjects.

There are studies indicate phenotypic differences of *Blastocystis* isolated from symptomatic and asymptomatic subjects (28, 30). Inter-and intra-subtypes variations were studied in the size and phenotype of *Blastocystis*. In this study, evaluation of phenotypical and size variations of *Blastocystis* was only performed on ST3 obtained from symptomatic and asymptomatic individuals. However, this variation might be related to the expression of antigens involved in pathogenicity of *Blastocystis* (28). Ragavan and colleagues assessed phenotypical and size variation of *Blastocystis* ST3 obtained from patients with gastrointestinal symptoms as well as patients who suffered from inflammatory bowel syndrome (IBS). They observed more variation in size and phenotypical of the parasite isolated from IBS patients and concluded that gut microbiota composition and intestinal environment, particularly in IBS patients, can lead to intra-subtype variation (30). In the current study, size variation was seen among all four studied subtypes of *Blastocystis* isolated from symptomatic and asymptomatic individuals. Size range of the subtypes in the current study was between 5 to 185 μm. Notably, ST1 showed the smallest size, followed by ST6, ST2, and ST3. However, although there is no comparative data of size and phenotype of common subtypes in human, it assumes that 1) widespread distribution of subtype 3 in environment, 2) broad spectrum sources, 3) probability of zoonotic transmission and 4) genetic diversity through inter-and intra-subtypes may be the main reasons of higher phenotypical and size variation of ST3 (31, 35). On the other words, since all studied subtypes in the current study are not host-specific, inter- and intra-subtype size variations more likely resulted from the source of infection.

The amoeboid form of *Blastocystis* was found only in a ST3 isolated from symptomatic subjects. This result is in line with previous studies showing correlation between the presence and suggested correlation between this stage with pathogenicity of the parasite (14). This finding was then supported by another study that indicated co-existence of acute urticarial associated with amoeboid forms of *Blastocystis* subtype 3 (26). However, amoeboid forms of *Blastocystis* have been mostly reported among ST3 obtained from symptomatic patients.

Interestingly, remarkable clumping of the parasite only seen in ST3 isolated from symptomatic subjects (Fig. 1). Previously, clumping of *Blastocystis* was reported from patients with IBS and/or other gastrointestinal symptoms (30). A probable scenario for this observation might be related to the ability of the pathogenic isolates of *Blastocystis* to colonize and explosive growth. Nevertheless, co-existence of clumping phenomenon of ST3 and some clinical manifestations were observed in *Blastocystis* reported from acute urticarial (26, 36) and the IBS patients with Blastocystis in Colombia (37).

Most of available studies have assessed growth profile of only one subtype that linked it to the physiological criteria of *Blastocystis* (28, 30). In this study, growth profile of 4 subtypes (ST1 –3 and 6) from symptomatic and asymptomatic patients was evaluated. In the current study, ST1 –3 and 6 from symptomatic patients and ST1 obtained from asymptomatic subjects showed higher rate of growth in comparison with other isolates. The exact reason of this observation is unclear, although the faster replication rate and higher number of the parasite at the peak of growth might be related to the greater pathogenicity of some isolates (not subtypes) in comparison with others. In the present study, the number of *Blastocystis* ST1 isolated from both symptomatic and asymptomatic subjects at the peak of growth was significantly more than other three subtypes. In addition, ST1 isolated from symptomatic and asymptomatic subjects showed smaller size in comparison with other isolates. The size of the parasite could be related to the rate of replication as well as the number of *Blastocystis* (30). On the other hand, there are studies stating a correlation between the presence of ST1 and some gastrointestinal symptoms (38, 39). However, the results showed explosive growth of *Blastocystis* among ST1 isolates from both symptomatic and asymptomatic subjects as well as those ST2 isolated from asymptomatic individuals. This finding indicates that not only there is no exact correlation between pathogenicity and ability of explosive growth of the parasite, but also this criterion could be originated from the features of *Blastocystis* subtype. Furthermore, in the studies conducted by Tan (28) and Ravagan (30), only one subtype (ST3) was evaluated in two symptomatic and asymptomatic groups, while in the current study, we investigated four different subtypes (ST1-3 and 6) in two symptomatic and asymptomatic groups. In other words, the analyses performed in this study was no comparison between two isolates (symptomatic/asymptomatic) from only one subtype (in the mentioned studies only ST3 was investigated). Therefore, differences in the results of our study in comparison with the mentioned works can be related to the more number of subtypes in two symptomatic and asymptomatic groups in our study. Reduced growth of the isolates at 96 h in our study could be resulted from outgrowth of bacteria even with presence of Penicillin-Streptomycin.

However, it needs to perform more works on the correlation between presence of this interesting eukaryote and acute/chronic clinical manifestations. Furthermore, studies that evaluate simultaneous association between inter- and intra-subtype genetic variation with size and phenotype of *Blastocystis* could be helpful.

## Conclusion

ST1 showed smaller size than other subtypes, while widest variation in phenotype and the size was seen in ST3. In addition, all ST3 in symptomatic patients were clumped and the amoeboid forms of *Blastocystis* only were found in a ST3 isolated from symptomatic subjects. Although explosive growth was recorded from only ST1 and ST2, there was no correlation between explosive growth and the presence of symptoms.
